# High-Quality Draft Genome Sequence and Annotation of the Basidiomycete Yeast *Sporisorium graminicola* CBS10092, a Producer of Mannosylerythritol Lipids

**DOI:** 10.1128/MRA.00479-19

**Published:** 2019-10-17

**Authors:** Stefany Solano-González, Alistair C. Darby, Doug Cossar, Mark X. Caddick

**Affiliations:** a Institute of Integrative Biology, The University of Liverpool, Liverpool, United Kingdom; b Croda Europe, Widnes, Cheshire, United Kingdom; University of California, Riverside

## Abstract

The basidiomycete Sporisorium graminicola (formally Pseudozyma graminicola) strain CBS10092 was originally isolated from an herbaceous plant in Russia. It is a known producer of mannosylerythritol lipids (MELs), the main component being MEL-C. Here, we present the 19.9-Mb draft genome sequence, which comprises 6,602 genes, including those encoding the MEL biosynthetic pathway.

## ANNOUNCEMENT

*Sporisorium graminicola* is a basidiomycete belonging to the Ustilaginomycetes (1). Members of this group, which includes the plant pathogen Ustilago maydis, produce a range of secondary metabolites with a wide range of potential industrial applications (2). Among these, biosurfactants are attracting particular attention as “green” alternatives to current commercial products (3–5). The principal biosurfactants produced by the Ustilaginomycetes group are the mannosylerythritol lipids (MELs), and *S. graminicola* has been reported to primarily produce MEL-C (6). Therefore, this strain has a potential application in the industrial production of MELs. However, little is known about this particular species. Here, we report the draft genome sequence of S. graminicola strain CBS10092, which was originally isolated from Plenum pratense in the Moscow region of Russia (1).

*S. graminicola* CBS10092 was grown in yeast extract peptone sucrose light (YEPSL) medium (7) for 48 h at 30°C in an orbital incubator (120 rpm). The harvested cells were homogenized using liquid nitrogen, and DNA was extracted by implementing the cetyltrimethylammonium bromide (CTAB) chloroform-isoamyl alcohol protocol (8). Genome sequencing was performed using the PacBio Sequel platform to generate 50× coverage with a total of 293,670 reads, which had an average read length of 13,690 bp. *De novo* assembly was performed with Hierarchical Genome Assembly Process (HGAP) version 3.2 (9), and contigs were ordered relative to the U. maydis genome as a reference using Mauve version 2.4.0 (10). This resulted in a chromosome-level genome assembly of 19.57 Mb, distributed in 22 contigs. The longest contig, at 2.35 Mb, was chromosome length. The assembly *N*_50_ value was 823,447 bp with a GC content of 56.75%.

Cells for RNA extraction were grown in a 5-liter fermenter containing 1 liter of medium (11) supplemented with 20 g/liter of glucose and enriched with fatty acid at a feed rate of 0.67 g/liter/h from 24 to 120 h. Cells were harvested by centrifugation and washed twice with phosphate-buffered saline (PBS). RNA was extracted using the RNeasy midi kit (Qiagen, Hilden, Germany) according to the manufacture’s protocol. To aid gene calling, strand-specific transcriptome sequencing (RNA-Seq) libraries were made from total RNA, using NEBNext poly(A) selection and Ultra directional RNA library preparation kits (New England BioLabs, Ipswich, MA, USA), and sequenced using the Illumina HiSeq 4000 platform. Illumina adapter sequences were removed from the FastQ files using Cutadapt version 1.2.1 (12). These were then trimmed using Sickle version 1.200 (13) with a minimum window score of 20. The Braker2 pipeline (14) was used to call genes and found a total of 7,190 transcripts, assigned to 6,602 genes.

Using the available annotation of *U. maydis* (15) and other related fungi (16–18), we annotated the *S. graminicola* genome and identified the MEL biosynthetic gene cluster. The *in silico* translation of the five genes from this cluster, *mac2*, *emt1*, *mac1*, *mmf1*, and *mat1*, shared 65%, 80.45%, 67.79%, 73.76%, and 56% identity, respectively, to the corresponding genes in *U. maydis*, and 76.64%, 56.49%, 61.96%, 74.28%, and 51.13% identity, respectively, to the corresponding genes in Moesziomyces aphidis DSM 70725. The *S. graminicola* sequence and annotation will aid in the utilization of this strain for future industrial exploitation.

### Data availability.

This whole-genome shotgun project has been deposited at DDBJ/ENA/GenBank under the accession number SRRM00000000. The version described in this paper is the first version, SRRM01000000 (as 22 contigs). All sequence data for RNA-Seq experiments used for annotation purposes have been submitted to the National Center for Biotechnology Information (NCBI) Sequence Read Archive (accession numbers SRR8919640 to SRR8919655). The raw reads for the PacBio sequencing can be found under the accession number SRR9845579.

## References

[1] Golubev W, Sugita T, Golubev N 2007 An ustilaginomycetous yeast, *Pseudozyma graminicola* sp. nov., isolated from the leaves of pasture plants. Mycoscience 48:29–33. doi:10.1007/S10267-006-0324-6.

[2] Hewald S, Linne U, Scherer M, Marahiel MA, Kamper J, Bolker M 2006 Identification of a gene cluster for biosynthesis of mannosylerythritol lipids in the basidiomycetous fungus *Ustilago maydis*. Appl Environ Microbiol 72:5469–5477. doi:10.1128/AEM.00506-06. 16885300 PMC1538720

[3] Sajna K, Sukumaran R, Jayamurthy H, Reddy K, Kanjilal S, Prasad R, Pandey A 2013 Studies on biosurfactants from *Pseudozyma* sp. NII 08165 and their potential application as laundry detergent additives. Biochem Eng J 78:85–92. doi:10.1016/j.bej.2012.12.014.

[4] Konishi M, Nagahama T, Fukuoka T, Morita T, Imura T, Kitamoto D, Hatada Y 2011 Yeast extract stimulates production of glycolipid biosurfactants, mannosylerythritol lipids, by *Pseudozyma hubeiensis* SY62. J Biosci Bioeng 111:702. doi:10.1016/j.jbiosc.2011.02.004. 21393057

[5] Kitamoto D, Hiroko I, Tadaatsu N 2002 Functions and potential applications of glycolipid biosurfactants—from energy-saving materials to gene delivery carriers. J Biosci Bioeng 94:187–201. doi:10.1016/S1389-1723(02)80149-9. 16233292

[6] Morita T, Konishi M, Fukuoka T, Imura T, Yamamoto S, Kitagawa M, Sogabe A, Kitamoto D 2008 Identification of *Pseudozyma graminicola* CBS 10092 as a producer of glycolipid biosurfactants, mannosylerythritol lipids. J Oleo Sci 57:123–131. doi:10.5650/jos.57.123. 18198469

[7] Lanver D, Tollot M, Schweizer G, Lo Presti L, Reissmann S, Ma L-S, Schuster M, Tanaka S, Liang L, Ludwig N, Kahmann R 2017 *Ustilago maydis* effectors and their impact on virulence. Nat Rev Microbiol 15:409–421. doi:10.1038/nrmicro.2017.33. 28479603

[8] Kim KE, Peluso P, Babayan P, Yeadon PJ, Yu C, Fisher WW, Chin C-S, Rapicavoli N, Rank DR, Li J, Catcheside DEA, Celniker SE, Phillippy AM, Bergman CM, Landolin JM 2014 Long-read, whole-genome shotgun sequence data for five model organisms. bioRxiv. 10.1101/008037. PMC4365909 25977796

[9] Chin CS, Alexander DH, Marks P, Klammer AA, Drake J, Heiner C, Clum A, Copeland A, Huddleston J, Eichler EE, Turner SW, Korlach J 2013 Nonhybrid, finished microbial genome assemblies from long-read SMRT sequencing data. Nat Methods 10:563–569. doi:10.1038/nmeth.2474. 23644548

[10] Darling A, Bob M, Blattner F, Perna N 2004 Mauve: multiple alignment of conserved genomic sequence with rearrangements. Genome Res 14:1394–1403. doi:10.1101/gr.2289704. 15231754 PMC442156

[11] Faria NT, Santos MV, Fernandes P, Fonseca LL, Fonseca C, Ferreira FC 2014 Production of glycolipid biosurfactants, mannosylerythritol lipids, from pentoses and d-glucose/d-xylose mixtures by *Pseudozyma* yeast strains. Process Biochem 49:1790–1799. doi:10.1016/j.procbio.2014.08.004.

[12] Martin M 2011 Cutadapt removes adapter sequences from high-throughput sequencing reads. EMBnet J 17:10–12. doi:10.14806/ej.17.1.200.

[13] Joshi NA, Fass JN (2011). Sickle: a sliding-window, adaptive, quality-based trimming tool for FastQ files (version 1.33). https://github.com/najoshi/sickle.

[14] Hoff KJ, Lange S, Lomsadze A, Borodovsky M, Stanke M 2016 BRAKER1: unsupervised RNA-Seq-based genome annotation with GeneMark-ET and AUGUSTUS. Bioinformatics 32:767–769. doi:10.1093/bioinformatics/btv661. 26559507 PMC6078167

[15] Kämper J, Kahmann R, Bölker M, Ma L-J, Brefort T, Saville BJ, Banuett F, Kronstad JW, Gold SE, Müller O, Perlin MH, Wösten HAB, de Vries R, Ruiz-Herrera J, Reynaga-Peña CG, Snetselaar K, McCann M, Pérez-Martín J, Feldbrügge M, Basse CW, Steinberg G, Ibeas JI, Holloman W, Guzman P, Farman M, Stajich JE, Sentandreu R, González-Prieto JM, Kennell JC, Molina L, Schirawski J, Mendoza-Mendoza A, Greilinger D, Münch K, Rössel N, Scherer M, Vraneš M, Ladendorf O, Vincon V, Fuchs U, Sandrock B, Meng S, Ho ECH, Cahill MJ, Boyce KJ, Klose J, Klosterman SJ, Deelstra HJ, Ortiz-Castellanos L, Li W, Sanchez-Alonso P, Schreier PH, Häuser-Hahn I, Vaupel M, Koopmann E, Friedrich G, Voss H, Schlüter T, Margolis J, Platt D, Swimmer C, Gnirke A, Chen F, Vysotskaia V, Mannhaupt G, Güldener U, Münsterkötter M, Haase D, Oesterheld M, Mewes H-W, Mauceli EW, DeCaprio D, Wade CM, Butler J, Young S, Jaffe DB, Calvo S, Nusbaum C, Galagan J, Birren BW 2006 Insights from the genome of the biotrophic fungal plant pathogen *Ustilago maydis*. Nature 444:97–101. doi:10.1038/nature05248. 17080091

[16] Lorenz S, Guenther M, Grumaz C, Rupp S, Zibek S, Sohn K 2014 Genome sequence of the basidiomycetous fungus *Pseudozyma aphidis* DSM70725, an efficient producer of biosurfactant mannosylerythritol. Genome Announc 2:e00053-14. doi:10.1128/genomeA.00053-14. 24526638 PMC3924370

[17] Konishi M, Hatada Y, Horiuchi J 2013 Draft genome sequence of the basidiomycetous yeast-like fungus *Pseudozyma hubeiensis* SY62, which produces an abundant amount of the biosurfactant mannosylerythritol lipids. Genome Announc 1:e00409-13. doi:10.1128/genomeA.00409-13. 23814110 PMC3695438

[18] Morita T, Koike H, Koyama Y, Hagiwara H, Ito E, Fukuoka T, Imura T, Machida M, Kitamoto D 2013 Genome sequence of the basidiomycetous yeast *Pseudozyma antarctica* T-34, a producer of the glycolipid biosurfactants. Genome Announc 1:e00064-13. doi:10.1128/genomeA.00064-13. PMC3622993 23558529

